# Supervised Resistance Training on Functional Capacity, Muscle Strength and Vascular Function in Peripheral Artery Disease: An Updated Systematic Review and Meta-Analysis

**DOI:** 10.3390/jcm10102193

**Published:** 2021-05-19

**Authors:** Elizabeth E. Blears, Jessica K. Elias, Christian Tapking, Craig Porter, Victoria G. Rontoyanni

**Affiliations:** 1 Department of Surgery, University of Texas Medical Branch, Galveston, TX 77555, USA; elizabeth.blears@ahn.org (E.E.B.); jeelias@utmb.edu (J.K.E.); christian.tapking@bgu-ludwigshafen.de (C.T.); cporter@uams.edu (C.P.); 2 Allegheny Health Network, Pittsburgh, PA 15212, USA; 3 Department of Pediatrics, University of Arkansas for Medical Sciences, Little Rock, AR 72202, USA; 4 Arkansas Children’s Nutrition Center, Little Rock, AR 72202, USA

**Keywords:** peripheral artery disease, claudication, resistance exercise, strength training, aerobic exercise, walking, functional capacity, muscle strength

## Abstract

Supervised resistance training appears to be a promising alternative exercise modality to supervised walking in patients with peripheral artery disease (PAD). This meta-analysis examined the efficacy of supervised RT for improving walking capacity, and whether adaptations occur at the vascular and/or skeletal muscle level in PAD patients. We searched Medline, CINAHL, Scopus, and Cochrane Central Register of Controlled Trials databases for randomized controlled trials (RCTs) in PAD patients testing the effects of supervised RT for ≥4 wk. on walking capacity, vascular function, and muscle strength. Pooled effect estimates were calculated and evaluated using conventional meta-analytic procedures. Six RCTs compared supervised RT to standard care. Overall, supervised RT prolonged claudication onset distance during a 6-min walk test (6-MWT) (101.7 m (59.6, 143.8), *p* < 0.001) and improved total walking distance during graded treadmill walking (SMD: 0.67 (0.33, 1.01), *p* < 0.001) and the 6-MWT (49.4 m (3.1, 95.6), *p* = 0.04). Five RCTS compared supervised RT and supervised intermittent walking, where the differences in functional capacity between the two exercise modalities appear to depend on the intensity of the exercise program. The insufficient evidence on the effects of RT on vascular function and muscle strength permitted only limited exploration. We conclude that RT is effective in prolonging walking performance in PAD patients. Whether RT exerts its influence on functional capacity by promoting blood flow and/or enhancing skeletal muscle strength remains unclear.

## 1. Introduction

Peripheral artery disease (PAD) is the third leading cause of cardiovascular morbidity, affecting more than 200 million people worldwide [1], and is the principal cause of non-traumatic lower limb amputation in the United States [2]. As a progressive atherosclerotic occlusive disease that primarily involves the lower limbs, PAD progressively reduces functional capacity [3] leading to mobility loss if left untreated [4]. Guideline-recommended therapies for PAD aim to lower cardiovascular risk, alleviate PAD symptomatology during claudication or critical limb ischemia, and to improve functional capacity [5]. While revascularization procedures to restore blood flow represent a frontline therapy to restore perfusion and ultimately function in patients with advanced PAD, supervised exercise therapy (SET) can improve functional capacity in PAD patients with claudication as well [6]. Early detection and diagnosis of PAD and adherence to guideline-recommended therapies including SET may even reduce the need for costly revascularization procedures and the risk for lower limb amputation. Indeed, since 2017, the Centers for Medicare & Medicaid Services cover SET for Medicare beneficiaries with symptomatic PAD [7].

A recent scientific statement from the American Heart Association on optimal exercise programs for PAD concluded that while treadmill-based supervised intermittent walking is the most studied mode of exercise with results consistently showing improved walking performance, other modalities, including resistance training (RT), may also be beneficial [6,8]. RT combined with aerobic training is highly indicated for the management of patients with chronic obstructive pulmonary disease (COPD) [9], a patient population that commonly suffers from peripheral muscle weakness and functional impairment. In fact, pooled evidence suggests that RT has similar effects on walking capacity, aerobic capacity (peak oxygen uptake), leg muscle strength and quality of life as endurance exercise in patients with COPD [10]. This observation suggests that RT may offer similar benefits in patients with PAD. While walking remains the recommended exercise modality in patients with PAD, there are conditions that act as barriers to engaging in walking, such as walking-induced pain (claudication), reduced walking capacity, foot ulcers, amputations or other comorbidities [8,11]. Alternative exercise modalities, such as RT, may act as a suitable substitute for PAD patients to deter from further functional decline and increased cardiovascular risk associated with prolonged sedentary behaviors [12], and even improve functional capacity and cardiovascular health. Indeed, a recent meta-analysis by Parmenter et al. [13] concluded that RT improves walking performance and appears to exert beneficial effects on muscle strength. Whether these changes in functional capacity are driven by adaptations at the vascular and/or skeletal muscle level remains unclear.

This systematic review and meta-analysis was undertaken to verify the efficacy of supervised RT for improving functional capacity in PAD patients and as an alternative modality to supervised walking/aerobic training (SupAer). A further objective was to examine whether adaptations occur at the vascular and/or skeletal muscle level in PAD patients in response to RT. We restricted our analysis to randomized controlled trials (RCTs) where supervised RT is the intervention group for PAD patients and is compared to SupAer or standard of care controls. Primary outcomes were changes in functional capacity as indicated by claudication onset distance (COD) and maximal walking distance on the 6-min walk test (6-MWT) or a progressive/graded treadmill test. Secondary outcomes were changes in potential mediators of improved functional capacity—namely, muscle strength and blood flow—where improved circulation to the lower extremities was indirectly indicated by changes in the ankle-brachial index [14], resting blood pressure, and vascular function, all acting as guides of therapeutic efficacy.

## 2. Materials and Methods

This systematic review and meta-analysis was registered in PROSPERO (CRD42019125505) on 4 April 2019 prior to data extraction and analysis.

### 2.1. Search Strategy

An author (E.E.B.), together with a research librarian at the University of Texas-Medical Branch, carried out the electronic study search in Pubmed/Medline, CINAHL, Scopus, and Cochrane Central Register of Controlled Trials databases including all searches from the earliest records until February 2019. Search terms included: ((exp Resistance Training) or resistance (train* or exercis*) or weightlift* or weight lift* or strengthen*(exercis* or program* or train*) or weight* bear*(exercis* or program*) or strength (train* or exercis* or program*) or bodybuild* or body build* or powerlift* or power lift* or theraband* or thera-band* or resistance (band or bands or tube or tubes or loop or loops) or medicine ball* or kettlebell* or kettle bell* or free weight* or dumbbell* or dumb bell* or weight machine* or strength machine* or deadlift* or (exp Weight Lifting)) AND (peripheral arter* disease* or exp Peripheral Arterial Disease). Language restrictions were not applied. Two authors (E.E.B., J.K.E.) independently screened retrieved abstracts after excluding duplicates, and three authors (E.E.B., J.K.E., V.G.R.) evaluated full-texts of potentially eligible studies to determine eligibility prior to inclusion. Two authors extracted the data independently (E.E.B., C.T.) and assessed trials for risk of bias (E.E.B., V.G.R.). Reference lists of relevant published systematic reviews and of included trials were scrutinized to identify any additional studies that database search terms may have missed.

### 2.2. Eligibility Criteria

Studies were included if they met the following criteria: (1) RCT design where intervention was supervised RT with length of ≥4 weeks in individuals diagnosed with PAD; (2) the study compared the effects of supervised RT against SupAer and/or standard of care controls. To be eligible for this review, RT must have included any type of resistance/strengthening exercises of the upper and/or lower body but not as part of circuit training. RT including a short warm up and cool down walking period was eligible. We excluded circuit type RT due to the aerobic component it introduces to the workout that could potentially confound the effect of RT. Aerobic-dominant exercise training was defined as training that involved “walking”, “treadmill”, “non-resistance cycling” or “pole-walking”. Studies using a combination of resistance and aerobic exercises as the intervention group were only eligible if they included an aerobic only comparator group which matched the aerobic component of the intervention group and that differed in no other parameter.

### 2.3. Exclusion Criteria

Trials were excluded if the primary outcome data had no relevance to cardiovascular function or functional capacity. Trials were also excluded if data represented combinations of patients with and without PAD where isolating data for the PAD group were not possible. Conference abstracts were included to minimize publication bias. Case reports, opinion articles, editorials and non-human studies were excluded. Duplicate publications generated from one RCT counted as one study. Studies were also excluded if the exercise intervention was not described in enough detail to confirm that RT occurred.

### 2.4. Risk of Bias

The Cochrane Collaboration’s risk of bias tool [15] was used to evaluate all included studies, and the original template was selected for compatibility with Review Manager 5.3 software (The Cochrane Collaboration, Copenhagen: The Nordic Cochrane Centre, Denmark). Quality assessment of all eligible papers was undertaken separately and in duplicate by two reviewers (E.E.B. and V.G.R.). Disagreements between the reviewers were resolved by consensus, or by a third reviewer if consensus could not be reached.

### 2.5. Outcome Measures

Primary outcomes were: COD, defined as the distance (in meters) walked up to the time claudication initiates on a graded treadmill test and/or 6-MWT; peak walking distance (PWD) and 6-MWT distance, defined as the maximal walking distance (in meters) achieved during graded treadmill testing and 6-MWT, respectively. If results were reported in time units (such as claudication onset time), these data were converted to meters by multiplying time units by the speed on the given treadmill test. Secondary outcomes were resting ankle-brachial index (ABI), muscle strength, resting brachial blood pressure, and vascular function. Resting ankle-brachial index (ABI), a simple diagnostic test for lower extremity PAD, is the ratio of ankle to brachial resting systolic blood pressure, where an ABI value of < 0.90 is indicative of PAD where the progressive reduction in ABI provides a measure of disease progression [5,14]. Vascular function is non-invasively and clinically assessed by a number of reliable methodologies with distinct characteristics and region of interest focusing on larger arteries (macrovascular function, including macrovascular endothelial function) or smaller resistance vessels (microvasculature function) [16]. Outcome measures screened for in the literature included flow-mediated dilatation of the brachial artery (FMD) for macrovascular endothelial function, pulse wave velocity as an index of aortic stiffness, pulse wave analysis as an index of pressure wave reflections, and venous occlusion plethysmography for microvascular function [16].

### 2.6. Data Extraction

Data were extracted by two investigators independently (E.E.B., C.T.) using a study collection form specifically developed for this systematic review per Cochrane guidelines [17]. Data extracted included study design, study population and setting, intervention details, outcomes, results, and publications information. If data was not available directly from the study report, a search of the clinical trials registry or other papers was made for relevant values regarding that study population. Data only presented graphically was extracted using Plot Digitizer 2.6.8 (plotdigitizer.sourceforge.net/) as recommended by the Cochrane Collaboration [17] and other published evidence [18]. Trials with duplicate data and/or multiple publications were grouped according to National Clinical Trial (NCT) identifiers (www.clinicaltrials.gov) or other clinical identifiers (Australia and New Zealand Clinical Trial Registry Identifier). For studies with multiple publications originating from the same trial population, data for demographics, study design and relevant outcomes were taken from the “index” article (i.e., the earliest publication reporting the trial’s study design, methods and maybe primary findings), and supplemented with additional outcomes data from later publications if relevant to the aims of this systematic review.

For results, detailed numerical data were collected, including sample size by group, absolute means and SD or 95% CI, mean change and SD or 95% CI of the change score, within-group and/or between group *p* values. Data were extracted for outcomes measured at baseline and at a follow-up nearest to the end of the intervention program.

### 2.7. Data Synthesis and Analyses

All outcomes were analyzed as post-intervention changes from baseline (mean difference between pre- and post- intervention data. Where change scores were not reported, these were calculated by subtracting baseline from post intervention values for each group separately. When the SD was not available, we estimated it from SEM or CI or IQR or ranges or actual *p* values [17,19]. Where SDs were obtained from *p* values, if levels of significance but no actual *p* values were reported, a conservative approach was undertaken where *p* < 0.05 is *p* = 0.049, *p* < 0.001 is *p* = 0.009, and *p* > 0.05 becomes *p* = 0.5. Inverse variance, random effects models were used on summary data from each intervention arm per study and pooled effect estimates (mean difference or standardized mean difference, and 95% CI) were calculated using Review Manager (RevMan version 5.3. Copenhagen, Denmark: The Nordic Cochrane Centre, The Cochrane Collaboration, 2014). Standardized mean difference was the selected summary statistic for outcomes measured on different treadmill protocols across studies and for muscle strength measures. Heterogeneity among studies was assessed using the I^2^ index. Forest plots were created to aid with visualization of the results. All data sets were assessed for normal distribution. Two-sided statistical significance was set at *p* < 0.05.

## 3. Results

### 3.1. Description of Studies and Exercise Interventions

**Figure 1:**
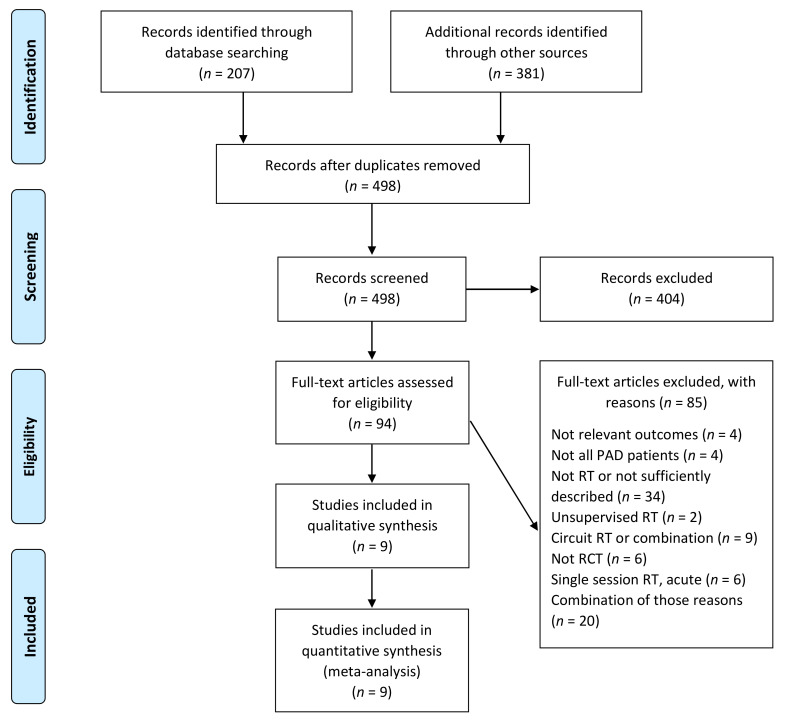
PRISMA flow diagram of the study selection process. PRISMA, Preferred Reporting Items for Systematic Reviews and Meta-Analyses; PAD, peripheral artery disease; RT, resistance training; RCT, randomized controlled trial.

After screening 498 unique and potentially relevant records identified from electronic databases and reference lists, 94 full texts were assessed for eligibility, and 15 articles [20,21,22,23,24,25,26,27,28,29,30,31,32,33,34], representing 9 RCTs, met the inclusion criteria. Six RCTs evaluated supervised RT compared to usual medical care (control group) [23,24,25,26,27,28,29,30], and five RCTs evaluated RT versus supervised aerobic exercise training [20,21,22,24,25,26,27,31,32,33,34], of which two RCTs examined all three study arms [24,27]. A PRISMA flow diagram [35] of the study selection process is detailed in Figure 1.

A pool of 467 participants was enrolled across the 9 included studies; 205 in RT, 162 in SupAer, and 100 in usual care/control group. Mean age of study participants ranged from 61 to 79 years, with ABI ranging from 0.52 to 0.74 across studies. The attrition rate for study participants ranged from 0 to 15% in six RCTs [20,22,27,28,29,31], reached 30% in one RCT (intention-to-treat analyses employed) [23] and was unclear/not specified in 2 RCTs [24,34]. Compliance to exercise intervention rates were clearly reported in four RCTs, ranging from 80 to 95% [20,22,27,30,31]. 

Length of the RT and SupAer interventions was 6 wk. in one RCT [30], 12 wk. in five RCTs [20,22,23,24,31,34], and 24 wk. in three RCTs [27,28,29]. Frequency of sessions was 2 times/wk. in three trials [23,31,34] and 3 times/wk. in six trials [20,24,27,28,29,30]. Exercise session duration ranged from 40 to 68 min in six trials [23,24,27,30,31,34] and was unclear or not reported in three trials [20,28,29]. RT involved exercises of the whole body in 5 trials [20,23,28,29,31], solely the lower limbs in 3 trials [24,27,34] and only the upper body in 1 trial [30]. The number of different exercises varied from 5 to 14, with 3 sets per exercise in the majority of trials, and repetitions per set ranging from 6 to 30. The characteristics of included studies and exercise interventions are presented in Table 1.

### 3.2. Risk of Bias of Included Studies

**Figure 2:**
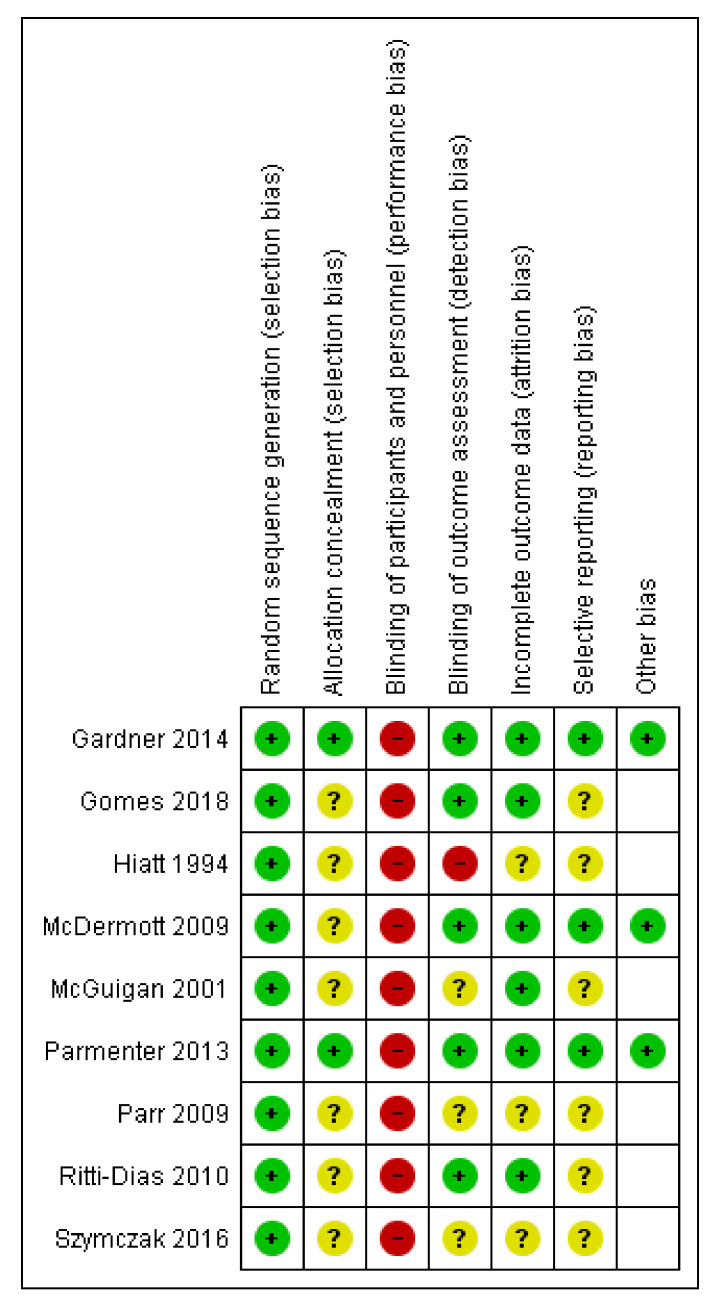
Risk of bias summary per included study [20,21,22,23,24,25,26,27,28,29,30,31,32,33,34].

Risk of bias is summarized in Figure 2. Trial quality varied across studies, which could overall be described as moderate. Information on allocation concealment was adequate in two RCTS [20,29] and unclear in the remaining seven. Due to the nature of the intervention (exercise training), only the outcome assessors were blinded to intervention assignment in five studies [20,23,27,29,31] and was unclear in the remaining four RCTs. Risk of attrition bias was considered low in 6 studies, in which attrition rate was either low [28,29,31] or intention-to-treat analyses [20,23,27] were employed, and unclear in the rest 3 RCTs [24,30,34]. As for risk of reporting bias, this was impossible to infer for the majority of trials.

### 3.3. Effect of RT on Walking Capacity 

**Figure 3:**
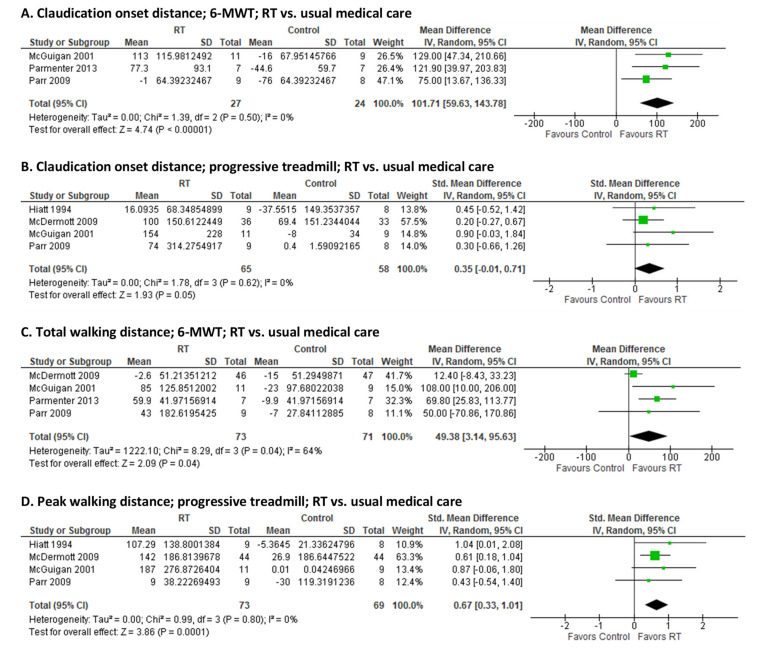
Pooled estimates and forest plots for supervised resistance training (RT) versus usual medical care on: claudication onset distance during a 6-min walk test (6-MWT) (**A**), progressive treadmill walking (**B**); total/peak walking distance during 6-MWT (**C**), progressive treadmill walking (**D**).

RT compared to standard of care: Six RCTs compared the effects of moderate-to-high intensity supervised RT to standard of care treatment in PAD. In three studies [28,29,30] that measured COD during a 6-MWT (I^2^ = 0%; Figure 3A), RT prolonged claudication onset for 101.7 m (59.6, 143.8, *p* < 0.001) over standard care. In four studies [24,27,28,30] that measured COD during progressive treadmill walking, RT increased COD compared to control (pooled SMD: 0.35 (−0.01, 0.71), *p* = 0.05; I^2^ = 0%; Figure 3B). 6-MWT distance [27,28,29,30] and PWD [24,27,28,30] during graded treadmill testing were reported in 4 studies, with RT significantly increasing walking distance covered by PAD patients during both tests (pooled MD_6-MWT_: 49.4 m (3.1, 95.6), *p* = 0.04; I^2^ = 64%; Figure 3C; pooled SMD_Treadmill_: 0.67 (0.33, 1.01), *p* < 0.001; I^2^ = 0%; Figure 3D).

**Figure 4:**
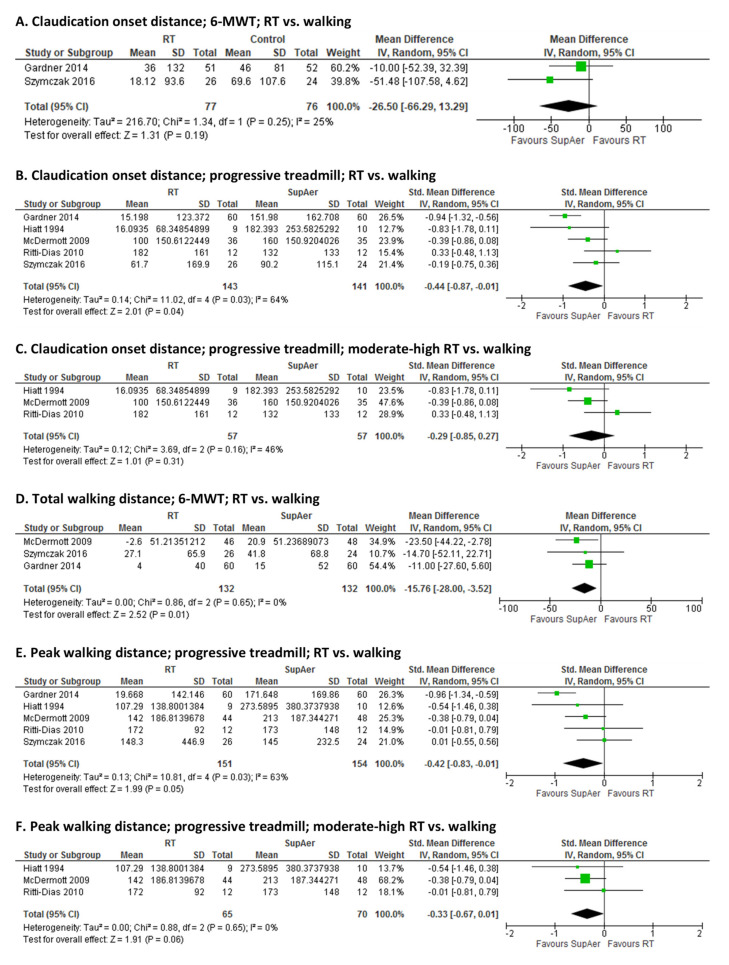
Pooled estimates and forest plots for supervised resistance training (RT) versus supervised walking on: claudication onset distance during a 6-min walk test (6-MWT) (**A**), progressive treadmill walking (**B**) progressive treadmill walking excluding mild intensity (**C**); total/peak walking distance during 6-MWT (**D**), progressive treadmill walking (**E**), progressive treadmill walking excluding mild intensity (**F**).

RT compared to SupAer: Five RCTs compared the effects of RT to SupAer (supervised walking), where RT ranged from light-to-moderate-to-high intensity across studies. Comparisons in graded treadmill COD between RT and SupAer were tested in all five studies [20,24,27,32,34], where SupAer was more beneficial (pooled SMD: −0.44 (−0.87, −0.01), *p* = 0.04; I^2^ = 64%; Figure 4B). Only two studies [22,34] reported COD during the 6-MWT, and hence the interpretation of those results need caution (pooled MD: −26.5 m (−66.3, 13.3), *p* = 0.19; I^2^ = 25%; Figure 4A). Summary estimates for 6-MWT distance showed greater effects of SupAer compared to RT alone based on three studies [20,27,34] (pooled MD: −15.8 m [−28.0, −3.5], *p* = 0.01; I^2^ = 0%; Figure 4D) and PWD during progressive treadmill testing based on five studies [20,24,27,32,34] (SMD: −0.42 (−0.83, −0.01), *p* = 0.05; I^2^ = 63%; Figure 4E). In order to check whether lighter intensity RT influenced our results, we performed sensitivity analyses where the two RCTS [20,34] using lighter intensity exercise were excluded from the pooled estimates for graded treadmill testing for COD and PWD. Neither test reached significance when only studies of moderate-high intensity were included instead (COD pooled SMD_Treadmill_: −0.29 (−0.85, 0.27), *p* = 0.31; I^2^ = 46%; Figure 4C; PWD pooled SMD_Treadmill_: −0.33 (−0.67, 0.01), *p* = 0.06; I^2^ = 0%; Figure 4F).

### 3.4. Effect of RT on Muscle Strength, Blood Pressure, ABI and Vascular Function

**Figure 5:**
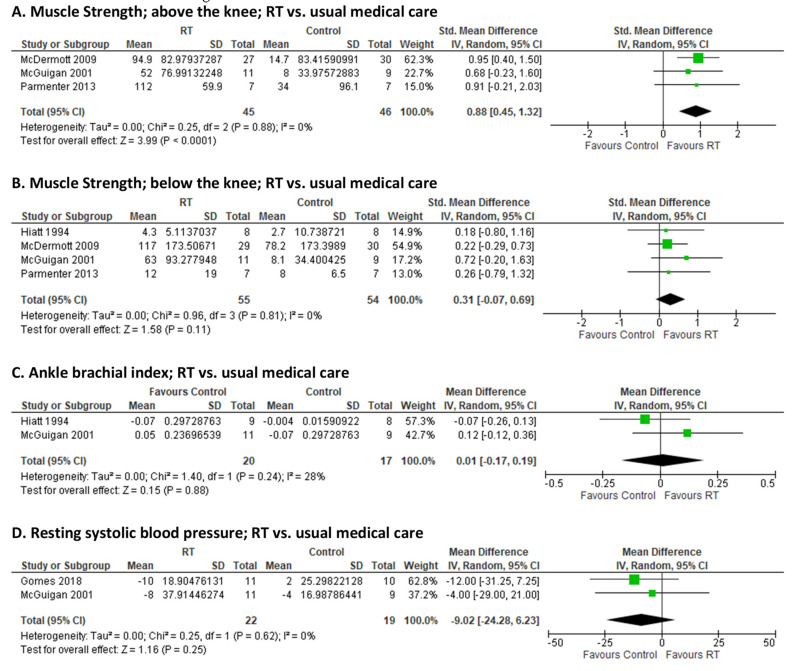
Pooled estimates and forest plots for supervised resistance training (RT) versus usual medical care on: muscle strength above the knee (**A**); muscle strength below the knee (**B**); ankle-brachial index (**C**); resting systolic blood pressure (**D**).

Pooled effect estimates from 3 RCTs [27,28,29] showed improved muscle strength of the upper leg in response to RT (SMD: 0.88 [0.45, 1.32], *p* < 0.0001; I^2^ = 0%; Figure 5A). Muscle strength of the lower leg did not significantly respond to RT based on pooled effect estimates from 4 RCTs [24,27,28,29] (Figure 5B). Supervised RT exerted no effect on ABI compared to usual care/control based on two studies [26,28] (I^2^ = 28%; Figure 5C). There was a decrease in resting systolic BP following RT based on summary estimates from 2 RCTs [23,28] but it did not reach statistical significance (pooled MD: −9.0 mmHg (−24.3, 6.2), *p* = 0.25; I^2^ = 0%; Figure 5D). A single RCT [23] from the included trials reported mean arterial pressure, hence no meta-analytic estimates could be produced. Regarding vascular function, one trial [27] tested the effects of supervised RT on endothelial function as measured by brachial artery FMD, one trial [20] reported arterial compliance as measured by arterial tonometry, and one trial [23] estimated systemic vascular resistance indirectly from cardiac output and mean arterial pressure. Due to the heterogeneity between the outcome methodologies for vascular function in these studies, no pooled estimates were generated.

## 4. Discussion

Given that PAD prevalence increases with advancing age and population aging is increasing globally, it is highly likely that the prevalence of PAD will continue to rise, placing significant burdens on health care systems [36]. Previous meta-analyses and current clinical guidelines have underscored the efficacy of exercise training in improving the walking capacity of PAD, especially of supervised treadmill walking therapy [6,8,37,38,39]. Although structured home-based exercise also improves walking performance in PAD patients [40], evidence suggests the effects of SET are superior [41]. The present systematic review and meta-analysis aimed to verify the efficacy of supervised RT for improving functional capacity in PAD patients and as an alternative modality to supervised walking/aerobic training, and to examine whether adaptations occur at the vascular and/or skeletal muscle level. Our work confirms the findings of a prior meta-analysis [13] by demonstrating that supervised RT improves the walking capacity of PAD patients, and at moderate to high intensity may offer an alternative to walking when that is not an option. Yet, the mechanisms that underpin the beneficial effect of RT on functional status, whether inducing adaptations at the vascular and/or skeletal muscle level, are poorly studied and cannot be ascertained based on the current evidence.

The 6 MWT and graded treadmill walking tests are clinical tools to accurately and reliably measure walking performance in response to SET in patients with PAD [42,43]. Yet, changes in 6 MWT are more readily interpretable across studies since pooled effect sizes remain in the original units of measure, whereas the heterogeneity of graded treadmill protocols requires mean differences from different studies to be normalized to SD which are clinically less meaningful [44,45]. In our meta-analysis, moderate-to-high RT ranging from 6 to 24 weeks prolonged the distance PAD patients were able to walk until claudication onset or maximal pain, with improvement in overall walking performance being more pronounced in response to the 6 MWT. Considering that PAD patients in the included RCTs could walk on average 140 m before they started experiencing leg pain during the 6 MWT, an increase of ~100 m in claudication onset time following RT that we demonstrated in our analysis is of great clinical importance.

To examine what potentially mediates the beneficial effects of supervised RT on walking capacity, we systematically reviewed the published literature for the effects of RT on muscle strength and vascular outcomes. As an atherosclerotic occlusive disease, the functional impairment associated with PAD originates from the blood flow limitation to the lower extremities and extends to structural and metabolic abnormalities in skeletal muscle [46,47]. Therapeutic strategies such as SET may target both the hemodynamic (systemic and local) and skeletal muscle tissue (local) components. Although SET alone cannot restore ABI, it has been shown to increase microvascular blood flow and oxygen utilization in the exercising skeletal muscle [48]. In agreement with other types of SET, our meta-analysis showed no changes in ABI following supervised RT, yet this analysis was just explorative due to the small number of studies included (2 RCTs [26,28]). Moreover, the drop in pooled estimates for systolic BP in response to moderate-to-high intensity supervised RT, while not statistically significant, may worth further examination in a well-powered RCT. While we systematically reviewed electronic databases to identify published records on the effects of RT on blood flow and vascular function, the evidence was sporadic and methodologies for vascular function assessment varied extensively between studies, precluding further meta-analytic evaluation. In a single RCT [27], macrovascular endothelium-dependent vasodilation, expressed as relative change in FMD [49], did not improve following 24 weeks of moderate-high supervised RT. Further, 12 weeks of light supervised RT did not affect large and small arterial compliance [20], and 12 weeks of moderate RT did not influence systemic vascular resistance [23]. Clearly, more research is needed to solidify whether or not there is an effect of RT on blood flow, BP and the vascular component, and whether changes in these outcomes are associated with changes in functional capacity following RT. At the local musculature level there seems to be more promising data, with our meta-analysis suggesting a significant increase in above the knee muscle strength following RT. Indeed, a SMD of 0.88 as that reported in our meta-analysis for upper leg muscle strength represents a large effect of RT, based on Cohen’s rule of thumb for interpreting SMD effect sizes [17,50]. Yet, the relationship between changes in muscle strength and concomitant increases in walking capacity could not be addressed in this report due to the rather small number of included RCTs.

Exercise modality has become a focus in clinical exercise science in terms of determining if one mode of exercise is more efficacious and/or more feasible. Accordingly, we examined how RT compares to supervised aerobic exercise, which for all identified and included studies was interval treadmill walking, consistent to expert guideline recommendations. Pooled estimates for walking capacity were greater following treadmill walking than RT, especially when performance was tested on the treadmill. One could argue that the treadmill walking group would be more accustomed to treadmill equipment and possibly outperform the RT group during treadmill testing assessment. This might have introduced bias in outcome measurement (treadmill testing for walking capacity) which would affect the outcome in a systematically different manner between walking and RT groups. Indeed, it has been previously demonstrated that greater improvements in functional capacity were achieved by patients who consistently trained on the same apparatus that was used in outcome assessments [51]. Hence, the effects of RT on walking capacity on the treadmill might have been underestimated compared to treadmill walking training. Furthermore, when light intensity RT was excluded from the analysis, treadmill walking training did not outperform RT on treadmill walking performance, suggesting that moderate-to-high intensity RT could act as an alternative training mode to supervised walking.

### Limitations and Future Research

Training regimens vary in session length, exercise intensity, number of exercises, repetitions and sets, area trained (upper or lower body) and session frequency, as well as the duration of the exercise intervention. Such heterogeneity is common in systematic reviews and meta-analyses of exercise training interventions. In addition, the rather limited number of included studies and of small sample size do not allow for firm conclusions to be made from the comparisons between RT and supervised treadmill walking as part of this meta-analytic work. Furthermore, the generalizability of our findings is limited to older symptomatic PAD patients, which are a fraction of the large population of PAD patients. Although patients were randomly allocated to study interventions in all included studies, imbalance between groups in prognostic factors such as patient comorbidities is possible. Future RCTs should be sufficiently powered to allow for adjustment of prognostic variables [52] and well-controlled by appropriately matching exercise intensities between RT and walking groups. For example, one study [20] described the intervention related to the resistance-regimen arm as attention control and this consisted of resistance exercises that were much lower in intensity than the supervised aerobic-dominant exercise arm. These lighter treatment regimens are likely to mask the benefits of RT, if any are present above those derived from aerobic-dominant exercise of greater intensity. Another concern that could be addressed in future studies is whether delayed onset of muscle soreness was present post-training and whether it interfered with adherence to the regimens. 

The evidence was even more restrictive in our explorations for the mechanistic pathways that explain the RT benefits on walking performance, whether these are driven by vascular and/or skeletal muscle adaptations. While great strides have been made to standardize walking tests for evaluation in PAD patients, this type of standardization has not taken place with tests of muscle strength for RT, and there is a plethora of vascular function methodologies assessing different regions and functions of the vasculature. Exploring ways to standardize the evaluation of skeletal muscle and vascular function, potentially by applying a combination of approaches within a single study, will improve the quality of the findings in this area and allow for comparisons between studies. Evaluation of different exercise regimens will also benefit from the assessment of muscle metabolic adaptations using near-infrared spectroscopy (NIRS) tissue oximetry [53]. In conditions that act as barriers to engaging in walking, such as foot ulcers or intense leg pain during walking, NIRS can offer a tool to trace any exercise-induced adaptations to foot or leg muscle perfusion and any effects on PAD severity [53,54]. 

PAD places a heavy burden on the domestic healthcare system, which affects 8–12 million US adults, results in approximately 70,000 new major amputations in the US per year [55], and adds up to annual hospitalization costs of more than $21 billion [56]. Understanding the ideal exercise regimen for the treatment of PAD, alone or in combination with surgical procedures, remains a high priority. 

## 5. Conclusions

Prior meta-analyses and expert guidelines have recognized the therapeutic potential of SET in maintaining activities of daily living and mobility in PAD patients. This review demonstrated that RT is effective in prolonging walking performance in PAD patients. Whether RT exerts its influence on functional capacity via reversing actions on the blood flow deficit and/or enhanced skeletal muscle strength remains unclear due to a limited number of published studies and the disparate outcome methodologies used. For PAD patients who are unable to participate in traditional supervised walking training due to ulcers or amputations or who feel insecure in their ability to walk on a treadmill, RT may provide an alternative that confers substantial benefit. Therefore, suitable resistance exercise regimens, perhaps incorporating inexpensive resistance bands, may be used to improve outcomes in a broader population of patients suffering from PAD.

## Figures and Tables

**Table 1:** Characteristics of the RCTs included in this systematic review and meta-analysis.

RCT		Group (*n*)	Mean Age (Years)	MeanABI	Supervised RT Group	Comparison Group	Session (Min)	RT Intensity	Set × Rep × Ex (*n*)	Sessions per Wk	Program Duration (wk)
	Supervised RT vs. Usual Medical Care Control										
Gomes 2018 (Brazil) [23]		RT (15) Control (15)	61 66	0.73 0.70	Whole body machine-based RT	Stretching and relaxation exercises	40	Moderate	3 × 10 × 8	2	12
Hiatt 1994 (US) [24,25,26]		RT (9) Control (10)	67 67	0.52 0.61	Lower limb isotonic free-weight RT	Usual medical care	60	Moderate-High	3 × 6 × 5/leg	3	12
McDermott 2009 (US) [27]		RT (52) Control (51)	72 69	0.62 0.60	Lower limb machine-based/BW RT	Attention control	40	Moderate-High	3 × 8 × 5	3	24
McGuigan 2001 (US) [28]		RT (11) Control (9)	70 69	0.61 0.67	Whole body machine-based/free-weights/BW RT	Usual medical care	NR	Moderate-High	2 × 8–15 × 8	3	24
Parmenter 2013 (AU) [29]		RT (8) Control (7)	79 71	0.53 0.55	Whole body machine-based RT	Usual medical care	NR	High	3 × 8 × 8	3	24
Parr 2009 (South Africa) [30]		RT (9) Control (8)	66 62	NR NR	Upper body machine-based/ free-weights RT	Usual medical care	45	Moderate	1 × 15–30 × 14	3	6
	**Supervised RT vs. Supervised Aerobic Training**										
Gardner 2014 (US) [20,21,22]		RT (60) SupAer (60)	65 65	0.74 0.68	Whole body machine-based RT	Treadmill walking	NR 15–45 min	Light	1 × 15 × 9	3	12
Hiatt 1994 (US) [24,25,26]		RT (9) SupAer (10)	67 67	0.52 0.55	Lower limb isotonic free-weights RT	Treadmill walking	60	Moderate-High	3 × 6 × 5/leg	3	12
McDermott 2009 (US) [27]		RT (52) SupAer (53)	72 72	0.62 0.60	Lower limb machine-based RT	Treadmill walking	40	Moderate-High	3 × 8 × 5	3	24
Ritti-Dias 2010 (Brazil) [31,32,33]		RT (15) SupAer (15)	66 65	0.63 0.66	Whole body machine-based RT	Treadmill walking	68	Moderate	3 × 10 × 8	2	12
Szymczak 2016 (Poland) [34]		RT (26) SupAer (24)	NR NR	0.70 0.67	Lower limb machine-based RT	Treadmill walking	50	Light-Moderate	3 × 15 × 6	2	12

RCT, randomized controlled trial; ABI, ankle-brachial index; RT, resistance training; rep, repetitions; ex, exercises; wk, week; BW, body weight; NR, not reported.
